# Autologous fibroblast therapy for facial rejuvenation: A randomized open-label controlled study

**DOI:** 10.1016/j.jpra.2026.05.045

**Published:** 2026-06-01

**Authors:** Pietro Gentile

**Affiliations:** Associate Professor of Plastic Surgery at the Department of Surgical Science, University of Rome Tor Vergata, 00173, Via Montpellier 1, Rome 00133, Italy

**Keywords:** Face rejuvenation, Fibroblasts, Regenerative medicine, Clinical trial, Plastic surgery

## Abstract

**Background:**

The aging process of the face is characterized by dermal thinning, loss of elasticity, and wrinkle formation. Injection of autologous micrografts containing fibroblasts (Am-FBs) has emerged as a promising regenerative technique.

**Objectives:**

This trial aims to assess the efficacy and safety of Am-FBs for facial rejuvenation.

**Methods:**

A randomized, open-label, controlled study was conducted. Forty patients presenting with mild to moderate dermal thinning, reduced elasticity, and wrinkles were divided into two groups: a study group (SG; n = 20) that received Am-FBs treatment and a control group (CG; n = 20) treated with a hyaluronic acid (HA)–based skin booster. Both groups received treatment at baseline (T0), at one month (T1), and at three months (T2). Clinical outcomes were evaluated at T1, T2, T3 (6 months), and T4 (12 months) through patient and physician evaluations, the Wrinkle Severity Rating Scale (WSRS), and objective skin elasticity metrics. *In vitro* evaluation of the Am-FBs and immunophenotypic characterization of the nucleated cell population contained in the Am-FBs suspension was performed.

**Results:**

At T4, the SG showed significantly greater improvements in wrinkle depth, skin elasticity, and WSRS score than the CG (*p* < 0.01). The physician’s evaluation showed scores ranging from 9 to 4 (*p* = 0.049), whereas patient self-assessments ranged from 9 to 5 (*p* = 0.039). Patient satisfaction was higher in the SG. The in vitro analysis documented 2.780.750 nucleated cells/ml in Am-FBs with a viability of 89.61% and immunophenotype: CD34%0.2, CD45%0.2, CD44%98.7, CD200%80.0, Cytokeratin 15%99.4.

**Conclusions:**

Am-FBs significantly improve signs of facial aging and present a safe option for skin rejuvenation.

## Introduction

The natural aging process leads to the degradation of the dermal extracellular matrix (ECM), decreased fibroblast activity, and a loss of skin elasticity.^1^ Traditional cosmetic procedures, such as hyaluronic acid (HA)-based skin boosters, provide temporary benefits, but autologous cell-based therapies offer a biologically driven regenerative approach. Autologous micrografts containing fibroblasts—cells (Am-FBs) isolated from a patient's skin can be reintroduced into the dermis to enhance ECM production, particularly collagen and elastin.^2^ Several studies have shown clinical benefits in nasolabial fold depth reduction and dermal thickness restoration using fibroblast injections.^3^^,^^8^ In the field of aesthetic regenerative medicine, one of the most important roles of non-surgical procedures is played by Platelet-Rich Plasma (PRP), which aims to improve skin quality for face rejuvenation purposes, as reported by the author.^4^^,^^5^ In this case, in several countries, according to local rules, it is mandatory to obtain approval from the transfusion service after the patient’s blood test screening, limiting the use to physicians with specific authorization and a consolidated clinical practice. At the same time, in the field of aesthetic surgical procedures, fat grafting and related stromal vascular fraction cells (SVFs) may improve the face’s soft tissue defects and skin texture.^5^^,^^11^ In this case, a specific background and consolidated clinical practice are required for the physician. For these reasons, the present study aimed to investigate the scientific data obtained from an easy and fast, nonsurgical, aesthetic regenerative procedure based on Am-FBs.

This trial seeks to evaluate the efficacy of Am-FB therapy in reducing signs of facial aging through a randomized, open-label, controlled design

## Methods

### Study design and ethical considerations

**Scheme 1:**
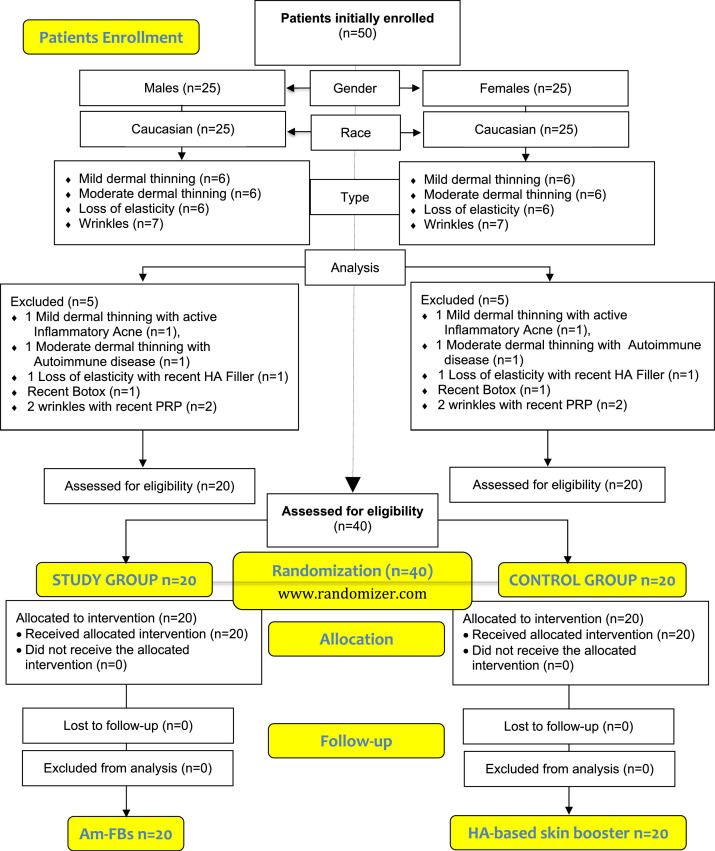
Consolidated Standards of Reporting Trials (CONSORT) flow diagram in patient enrollment.

A prospective randomized open-label controlled trial was performed in accordance with the Declaration of Helsinki and internationally recognized ethical guidelines for clinical research.^6^ Study quality was assessed using the Consolidated Standards of Reporting Trials (CONSORT) guidelines^7^ (http://www.consort-statement.org) (Scheme 1), and checklist (Supplemental Material 1). Written informed consent, reporting alternative strategies, side effects, complications, and recommendations, was obtained from all participants. No participant incurred any treatment-related costs. All procedures, devices, and materials used in the study were fully supported through the investigator’s personal research funding. The study was conducted exclusively for scientific and research purposes, without additional costs for participants. To avoid interference with outcome evaluation and follow-up standardization, no crossover or additional treatments were performed during the study period. All procedures were carried out in compliance with European regulations established by the European Medicines Agency (EMA), the Committee for Advanced Therapies (CAT) (EMA/CAT/600280/2010 Rev 1), and European Parliament Regulation (EC) No. 1394/2007. The procedure involved the use of Am-FBs obtained through a mechanical filtration process without cell expansion. In accordance with EMA Committee for Advanced Therapies (EMA-CAT) recommendations (Guideline on Human Cell-Based Medicinal Products, 2015, section 2.2.4a), this approach qualifies as a “*minimal manipulation”* technique, since the processing does not alter the biological characteristics, physiological functions, or structural properties of the cells. Consequently, the preparation—being used for the same essential function and without substantial manipulation—does not fall under the definition of an Advanced Therapy Medicinal Product (ATMP). The study protocol was formulated in accordance with the associate professor contract no. 13489/2021 between the author and the University of Tor Vergata, Rome, Italy, and as part of a research project approved by the Department of Surgical Sciences (approval no E83C22001960005).

### Patient enrollment

**Fig. 1:**
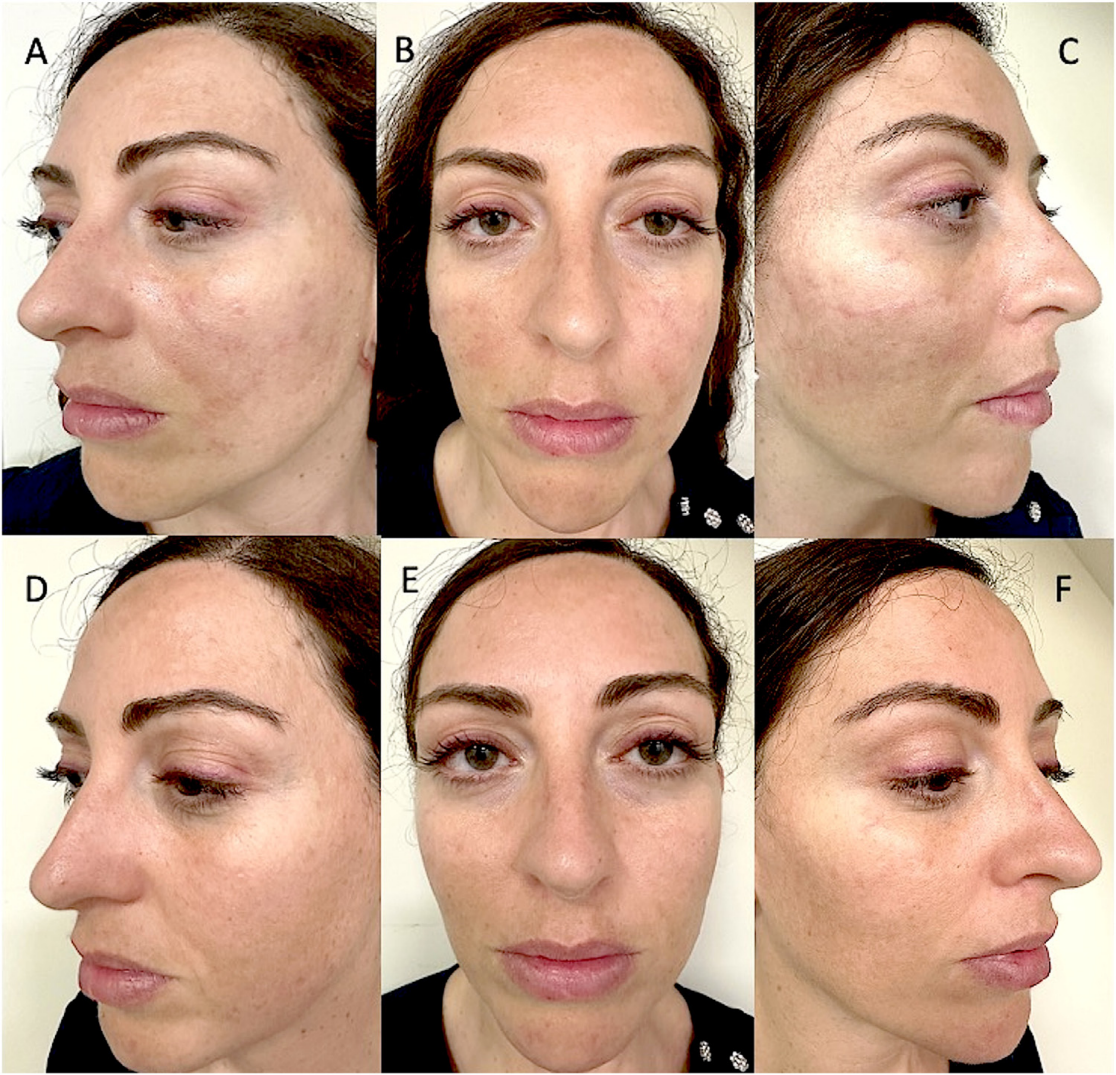
A 29-year-old female affected by moderate dermal thinning, loss of elasticity, and wrinkles, treated with Am-FBs. (A) Pre-operative projection in ¾ left view. (B) Pre-operative projection in frontal view. (C) Pre-operative projection in ¾ right view. (D) Post-operative projection after 6 months (T3) in ¾ left view. Improvement of the skin texture, tissue elasticity, and brightness, with reduction of wrinkles. (E) Post-operative projection in frontal view, evidencing all the improvements. (F) Post-operative projection after 6 months in ¾ right view.

Between January 2024 and January 2025, 40 patients with mild-to-moderate facial aging were enrolled. 20 patients with mild to moderate dermal thinning, reduced elasticity, and wrinkles were assigned to the study group (SG) and treated with Am-FBs (Fig. 1A–C). The SG included 10 women and 10 men aged 25–45 years (mean age: 35 years), with all female participants being premenopausal. Outcomes were compared with those of a control group (CG) consisting of 20 patients who received a hyaluronic acid (HA)–based skin booster Yfor the same indications (Supplementary Fig. 1A–C). The CG also comprised 10 women and 10 men aged 25–45 years, with all female participants premenopausal. No significant differences in baseline characteristics were observed between the two groups. All participants in both groups underwent comprehensive preoperative evaluation, including detailed medical history (with assessment of patient expectations), complete clinical examination, and documentation through standardized photography and the Wrinkle Severity Rating Scale (WSRS). Post-treatment follow-up assessments were conducted at T1, T2, six months (T3), and 12 months (T4).

### Clinical appraisals

The clinical appraisals were reported in a CONSORT flow diagram (Scheme 1). Data on patients (age, gender, race, and types of facial defects), intervention/comparator, outcomes, study design, exclusion criteria, and inclusion criteria were described according to the PICOS approach and detailed in Table 1.

**Table 1:** Study assessment based on inclusion and exclusion criteria according to the PICOS (patients, intervention, comparator, outcomes, and study design) approach (https://ro.ecu.edu.au/cgi/viewcontent.cgi?referer=https://www.google.it/&httpsredir=1&article=1010&context=ecupres).

	*Inclusion Criteria*
**P**-Patients	age 20–50 years, caucasians, patients with mild to moderate dermal thinning, loss of elasticity, wrinkles, facial soft tissue defects, facial skin aging, signs of facial aging
**I**-Intervention	Local injection of Am-FBs
**C**-Comparator	Skin booster with vitamines
**O—**Outcomes	*Clinical outcomes measures* Primary Outcome: Improvement in wrinkle severity based on Wrinkle Severity Rating Scale (WSRS). Global assessment scales performed by the physician and the patients Secondary Outcomes: Dermal elasticity via Cutometer® analysis. Patient-reported outcomes using a visual analog scale (VAS) for face appearance and satisfaction. Safety monitored via adverse event reporting and physical examination Tertiary Outcomes: Information about the risks and side effects. *Instrumental outcomes measures* *In vitro* presence of nucleated cells in the Am-FBs solution, *In vitro* quantitative (cell amount) and qualitative (live and dead cells) analysis of the cell population contained in Am-FBs solution using a flow cytometer. Fibroblast cell immunophenotyping.
**S**-Study Design	Randomized open-label case-control clinical trial

	*Exclusion Criteria*
**P**-Patients	Other types of defects, use of pharmacological therapeutics against facial skin aging as Platelet-Rich plasma (PRP), radiofrequency, micro-needling, filler, hyaluronic acid (HA), botox, active inflammatory acne, auto-immune diseases, uncompensated diabetes, sepsis, cancer.
**I**-Intervention	Platelet-Rich plasma (PRP), radiofrequency, micro-needling, filler, hyaluronic acid, botox.
**C**-Comparator	Not applied
**O—**Outcomes	Not applied
**S**-Study Design	Not applied

### Randomization and blinding

SG and CG participants were randomly assigned, based on the inclusion and exclusion criteria, to one of two treatment groups: SG (n = 20) or CG (n = 20). Randomization was carried out using a computer-generated random sequence (https://www.randomizer.org), with allocation concealed by a central randomization system. The study was open-label, meaning that both the participants and the investigators were aware of the treatment assignment.

### Clinical outcomes measures

Clinical outcome measures were categorized into three levels: primary, secondary, and tertiary. A global assessment scale was used, incorporating both objective and subjective evaluations. Patients completed the subjective assessment themselves, while the physician conducted the objective evaluation. The investigators’ assessment relied on clinical judgment and detailed image analysis, utilizing a six-point scale: [9] excellent, [8] very good, [7] good, [6] enough, [5] dissatisfied, and [4] very dissatisfied. This same six-point scale (from [9] excellent to [4] very dissatisfied) was also used by patients for self-assessment via a questionnaire (Supplemental Material 2 – Appendix A). Additional outcome variables included itching, slight redness, and mild numbness in the treated area.

Primary Outcome:

• Improvement in wrinkle severity based on WSRS.
• Overall evaluation scales completed by both the physician and the patients (Supplemental material 2 – Appendix A).

Secondary Outcomes:

• Dermal elasticity via Cutometer® analysis and patient satisfaction surveys.
• Patient-reported outcomes using a visual analog scale (VAS) for face appearance and satisfaction.
• Safety is monitored via adverse event reporting and physical examination

Tertiary Outcomes:

• Information about the risks and side effects.

### Clinical data assessment

The face soft tissue defects type assessment, treatment management, and follow-up were prospectively recorded in Scheme 1. All treatment options were reviewed and determined by a multidisciplinary team comprising a plastic surgeon and a dermatologist. The follow-up was performed by clinical examination, photography, and WSRS assessment. Any abnormal clinical findings were further evaluated as necessary. Data analysis was performed by independent evaluators who were not involved in patient treatment. Side effects and or complications were documented by clinical examination and anamnesis.

Although clinical follow-up examinations were performed by the treating physicians for procedural consistency and safety monitoring, image analysis and data evaluation were conducted by independent evaluators who were not involved in patient treatment. Nevertheless, the absence of fully blinded external evaluators for all outcome assessments remains a limitation of the study.

### Instrumental outcomes measures

Instrumental measures were performed using a flow cytometer, aiming to support this clinical study with in vitro data.

• To demonstrate in vitro the presence of nucleated cells in the Am-FBs solution,
• To perform in vitro a quantitative (cell amount) and qualitative (live and dead cells) analysis of the cell population contained in the Am-FBs solution.
• To perform immunophenotypic characterization of the nucleated cell population contained in the Am-FBs suspension.

### *In vitro* assessment

The in vitro assessment was conducted on 2-mm punch biopsies obtained from the retro-auricular/mastoid region, and the resulting A-FB suspension was mildly treated with ACK lysis buffer to get rid of red blood cells. Afterward, a viability dye (7-AAD) was added to the sample and analyzed by a flow cytometer (NovoCyte 451200533575, software NovoExpress 1.6.2). By using the FSC-SSC (size vs. granularity) plot nucleated cells were identified. The dead-live cell discrimination was performed from the “Nucleated cells” gate using the 7-AAD channel. Immunophenotypic measurements were performed using Beckman Coulter DxFLEX Flow Cytometry system, and CyExpert DxFLEX software program was used for evaluation. The procedures were performed by two biologists.

### Treatment protocol

**Fig. 2:**
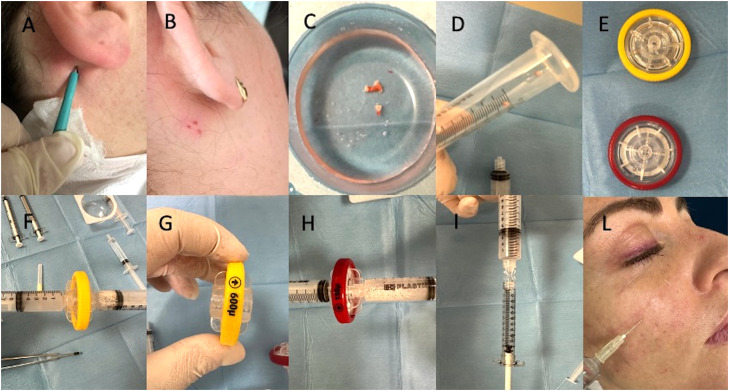
Am-FBs preparation procedure, according to the minimal manipulation rules using the Dermomine® t-lab kit (t-lab Esentepe Mah. Büyükdere Cd. No:151 Yonca Apt. C Blok No:42 Şişli, İstanbul https://tlab.com.tr/en/). (A) Punch biopsies of 2 mm were performed in the mastoid area. (B) 3 holes of 2 mm released by punch biopsies. (C) The three punch biopsies were collected in 10 mL of saline solution. (D) After the three-punch biopsies were collected in 10 mL Luer-Lok syringes, containing 8 mL of saline solution. (E) Above the 600-micron filter – yellow filter, and below the 150-micron filter – red filter, through the suspension was filtered (respectively, 51 passages into the yellow filter and only once passage into the red filter). (F) Filtration phase using a yellow filter (51 passages). (G) Detail of 600-micron filter; the arrow indicates the direction of filtration. (H) Filtration phase using a red filter (only once passage following the arrow direction). (I) 1 ml Luer-Lok syringes charged with A-FBs solution. (L) 6 mL of solution containing A-FBs was obtained. 0.2 mL of Am-FBs for each cm^2^ was injected into the selected area of the face through a nappage technique using a 1 mL Luer-Lok syringe.

Am-FBs were prepared according to the “*minimal manipulation”* rules using the Dermomine® t-lab kit (t-lab Esentepe Mah. Büyükdere Cd. No:151 Yonca Apt. C Blok No:42 Şişli, İstanbul https://tlab.com.tr/en/ continued in D MED GRUP MEDİKAL, Üçevler Mah. 70. Sk. İbrahim Yazıcı Plaza-1 No:1/22d Nilüfer, Bursa Turkey). Local anesthesia, 5 ml of lidocaine with adrenaline, was injected in the retroauricular area in the mastoid portion. 3 punch biopsies of 2 mm were performed in SG patients in the mastoid area (Fig. 2A, B). The punch biopsies were collected in 10 mL of saline solution (Fig. 2C) and immediately after into a 10 mL Luer-Lok syringe (Fig. 2D) containing 8 mL of saline solution. The syringe was filtered through a 600-micron filter (Fig. 2E, yellow filter) with 51 passages (Fig. 2F) in a specific direction (Fig. 2G, arrow). The filtered suspension obtained was filtered only once through a 150-micron filter (Fig. 2H, red filter), aiming to obtain a solution containing Am-FBs (Fig. 2I). 6 mL of solution containing Am-FBs was obtained. 0.2 mL of Am-FBs for each cm^2^ was injected into the selected area of the face through a nappage technique using a 1 mL Luer-Lok syringe (Fig. 2L).

HA-based skin booster was obtained from a commercially available HA-based skin booster (NCTF-135HA, Filorga, Paris, France https://fillmed.com/revitalize-nctf/), and the treatment was performed by injecting 0.2 ml of HA-based skin booster for each cm^2^ using the same nappage technique in the selected area of the face.

Both groups received treatment at baseline (T0), at one month (T1), and at three months (T2). Clinical outcomes were evaluated at T1, T2, T3, and 12 months (T4) using the WSRS and objective skin elasticity metrics. Exclusion and inclusion selection criteria were described in Table 1.

### The risk mitigation measures

The Risk Evaluation and Mitigation Strategies (REMSs) incorporating Elements to Assure Safe Use (ETASU) were applied in Am-FBs and HA-based skin booster treatments, significantly reducing safety-related risks. Key aspects of ETASU included the consistent application of a standardized treatment protocol for all patients, clearly defined inclusion and exclusion criteria, and the exclusive use of CE-marked medical devices and HA-based skin booster. All potential risks were assessed before initiating treatment, with the most common issue being unsatisfactory outcomes. REMSs generally emphasize proper training for healthcare professionals and thorough patient assessment. The main objective of ETASU was to ensure patient safety during access to these procedures. The authors investigated specific ETASU components through the FDA’s REMS database (http://www.accessdata.fda.gov/scripts/cder/rems/index.cfm) and identified the following elements:

• Informed consent form for all participants detailing potential side effects and risks;
• A structured training program for healthcare providers;
• A communication strategy regarding possible side effects;
• The requirement for CE certification for medical devices and HA-based skin boosters used in treatments;
• Patient enrollment is based on predefined inclusion and exclusion criteria.

### Statistical analysis

Statistical analyses were performed using SPSS software version 22.0 (IBM Corp., Armonk, NY, USA). Baseline characteristics were summarized using descriptive statistics. Continuous variables were expressed as mean ± standard deviation (SD). Within-group comparisons were performed using paired *t*-tests, while between-group comparisons were assessed using independent *t*-tests. A p-value < 0.05 was considered statistically significant.

## Results

### Clinical assessment

All procedures, including Am-FB treatment and the HA-based skin booster, were completed in every patient in both the study group (SG) and the control group (CG). Follow-up assessments were conducted at T1, T2, T3, and T4. All participants in both groups were evaluated at T4, marking the completion of follow-up.

### Primary outcome

At 12 months (T4), the SG treated with Am-FBs demonstrated a significant improvement in the WSRS score compared to the CG treated with HA-based skin booster (mean improvement: 1.4 points in the SG vs. 0.3 points in the CG, *p* < 0.01).

With respect to the physician’s assessment, scores ranged from 9 to 4 (*p* = 0.049). 14 SG patients (70%) (7 males and 7 females) and 10 CG patients (50%) (5 males and 5 females) were fully satisfied regarding the facial appearance (Fig. 1D–F and Supplemental Fig. 1D–F).

With respect to the patient’s evaluation, scores ranged from 9 to 5 (*p* = 0.039). 16 SG patients (80%) (8 males and 8 females) and 11 CG patients (55%) (6 males and 5 females) were fully satisfied regarding the facial appearance. The results indicate that SG patients were more satisfied compared to CG. No statistically significant differences between male and female participants were observed.

### Secondary outcomes

Cutometer measurements showed a 27% increase in skin elasticity in SG patients, compared to a 5% increase in the CG patients (*p* < 0.01).

The SG exhibited greater reductions in wrinkle visibility (mean change: 23% vs. 11%, *p* < 0.05) and higher patient satisfaction ratings (mean VAS score: 7.2 vs. 4.7, *p* < 0.05).

Adverse events were rare, and no significant differences were observed between SG and CG. The most common adverse event, reported by 3 SG patients and 3 CG patients respectively, was mild swelling and minor erythema at the injection sites, which resolved within 4 days.

### Tertiary outcomes

Analysis of the satisfaction questionnaire indicated that all participants in both groups (SG and CG) would opt for facial biorivitalization again and reported being adequately informed about the potential risks and complications of the procedures, including the possibility of needing repeated treatments and the risk of ineffectiveness (Supplemental Material 2 – Appendix A).

### *In vitro* outcomes

Flow cytometer analysis (Supplemental Fig. 2A, B) confirmed the presence of nucleated cells in Am-FBs solution (Supplemental Fig. 2A). The in vitro quantitative analysis documented a concentration of 2.780.750 nucleated cells/ml. The viability of the cells was 89.61% (Supplemental Fig. 2B), and the concentration was 2.491.950 cells/ml. Dead cells were 10.39% (Supplemental Fig. 2B). Immunophenotypic measurements showed the following results: CD34%0.2, CD45%0.2, CD44%98.7, CD200%80.0, Cytokeratin 15%99.4.

### Limitations and strengths

The five main limitations of the study were: (a) its open-label design, (b) the individualized treatment approach, (c) the limited sample size, (d) the relatively short follow-up period, and (e) the lack of granularity in statistics.

(a) The “open-label” design, rather than a single- or double-blind approach, limits the objectivity of the evaluation, as assessments could potentially be influenced by participants’ or investigators’ awareness of the treatment received. The lack of blinding introduces strong bias, particularly in patient-reported outcomes and physician assessments.
(b) Conversely, potential bias was partially reduced by the individualized, “custom-made” approach applied to each patient in both groups (SG and CG), with the volume of solution administered tailored to the specific type of defect.
(c) The relatively small sample size does not permit a definitive description of the Am-FB’s efficacy, not allowing the identification of any subgroups that could respond differently to the treatment. Additionally, only 20 patients per arm limits statistical power and generalizability. The paper lacks a power calculation.
(d) The limited follow-up based on a maximum of 12 months may not be enough to demonstrate the long-lasting results.
(e) Statistical results lack granularity (e.g., SD/SE, CI, full p-values). WSRS improvements are described, but details on scale validation and minimal clinically important difference (MCID) are missing.

Additionally, the study could not be performed in a double-blinded fashion because the procedures differed substantially between groups. Patients treated with Am-FBs underwent retroauricular punch biopsies for autologous tissue harvesting and immediate mechanical processing, whereas control patients received only HA-based injections. Therefore, both participants and treating physicians were inevitably aware of treatment allocation. To minimize potential bias related to the open-label design, standardized photography, WSRS scoring, Cutometer® measurements, predefined inclusion/exclusion criteria, and independent data analysis were adopted.

The main strengths of the study include the randomized controlled design, the translational approach combining clinical and in vitro analyses, and the use of objective instrumental measurements

Additionally, data analysis was performed by independent evaluators who were not involved in patient treatment.

## Discussion

This study supports previous research demonstrating the regenerative potential of Am-FBs for facial rejuvenation.^3^^,^^8^ Our results align with follow-up studies that report stable clinical improvement and no major adverse tissue reactions.^9^ Although the study is limited by its small sample size and open-label design, the results offer consistent preliminary evidence supporting Am-FBs therapy as an effective cosmetic treatment. Facial rejuvenation commonly involves a combination of aesthetic medical treatments to counteract the effects of aging, such as wrinkles, dermal and skin thinning, loss of elasticity, and volume depletion. As people age, collagen fibers—which provide structural support—become fragmented, and the connections between fibroblasts weaken.^10^ This results in skin sagging, reduced skin thickness, and loss of youthful characteristics. HA-based skin booster and vitamins help restore a youthful look. Synthetic fillers, including HA, calcium hydroxyapatite, polylactic acid, and polymethylmethacrylate, have bio-stimulatory properties that range from modest stimulation of fibroblast activity to more significant effects on skin thickness and blood flow.^10^ Between these products, in the present study, the HA-based skin booster was selected. For this reason, the author decided to use NCTF-135 HA Filorga. On the other hand, the emerging regenerative strategies can be useful to improve the signs of aging. PRP and fat grafting are effective methods for facial rejuvenation; they are autologous, natural, and have regenerative benefits. The use of autologous PRP or fat grafting in face rejuvenation has already been demonstrated by the author of the present paper.^4^^,^^11^ About the PRP use, in several countries, according to local rules, it is mandatory to obtain approval from the transfusion service after the patient’s blood test screening, limiting the use to physicians with specific authorization and a consolidated clinical practice.^4^ The PRP efficacy has also been documented through randomized double blind controlled studies, as performed recently by Tsai YW et al.,^13^ and previously by Alam M, et al.^14^ In the paper published by Tsai YW et al.,^13^ PRP demonstrated greater effectiveness in improving overall skin quality in patients affected by facial photaging compared to platelet-poor plasma (PPP).^13^ In the study by Alam et al.,^14^ participants reported that both fine and coarse skin texture showed significantly greater improvement after a single PRP treatment compared to normal saline. About the fat grafting and the related SVFs, it is necessary to have a specific background and consolidated clinical practice to perform this surgical strategy. Additionally, according to local rules, it is mandatory to respect the minimal manipulation rules.^11^ Recently, Zhu Y et al.^15^ compared the effectiveness of SVF-gel transplantation with a combination of nano-fat and high-density fat prepared using the Coleman technique (nano-fat + high-density fat) for periorbital volume restoration and rejuvenation in early periorbital aging. Both SVF-gel and nano-fat + high-density fat were effective in restoring periorbital volume and improving early signs of aging. However, patients treated with SVF-gel had a significantly lower reoperation rate and higher satisfaction scores compared to those who received nano-fat + high-density fat. Exosomes, particularly those derived from adipose-derived mesenchymal stem cells (ASCs), appear to play a promising role in facial rejuvenation, as recently reported by Estupiñan B et al.^12^ Exosomes, an emerging therapeutic option in aesthetic medicine, have applications in skin rejuvenation, alopecia, atopic dermatitis, acne scarring, and wound healing.^12^ In an investigator-blinded, split-face study, Estupiñan B et al.^12^ compared the safety and efficacy of ASC-derived exosomes with PRP for photoaged facial skin. Both treatments similarly improved wrinkling, dyschromia, erythema, texture, and overall skin appearance. Histological analysis showed increased collagen I and glycosaminoglycans in both groups, with no significant differences between them. Estupiñan B et al.^12^ concluded that ASC exosomes represent a promising alternative to PRP, particularly for needle-averse patients, and offer the added benefit of shorter office visits since phlebotomy and centrifugation are not required. In the same way, also in the present study, based on the results obtained, both Am-FBs and HA-based skin boosters appear as two valid and safe strategies to perform face rejuvenation and skin biorevitalization. Am-FBs are an emerging regenerative strategy that may be attractive for patients needing more effective treatment, and, at the same time, they must be available to undergo some punch biopsies in the mastoid area. Starting from this point, a new study could be the comparison between two different autologous regenerative not-surgical strategies: PRP and Am-FBs (Table 2).

**Table 2:** Patient satisfaction data.

	Study Group (SG)	Control Group (CG)
Patients no°	20	20
Self-evaluation of cosmetic results (score range 4–9 / excellent-very dissatisfied)	**16 (Fully Satisfied)**:	**11 (Fully Satisfied)**:
	8 (Excellent/9)	3 (Excellent/9)
	4 (Very good/8)	4 (Very good/8)
	4 (Good/7)	4 (Good/7)
	4(Not/Fully/Satisfied):	5(Not/Fully/Satisfied):
	4 (Enough/6)	5 (Enough/6)
		4(Not/Satisfied):
		4 (Dissatisfied/5)
		0 (Very Dissatisfied/4)
Satisfaction of result	20	16
Available to next treatment	20	16
Recommend face biorivitalization to a friend	20	16
Sufficiently informed about risks and complications	20	20
Available to do face biorivitalization	20	20

## Conclusions

Am-FB’s therapy significantly improves facial wrinkle severity and skin elasticity, with minimal adverse effects. This cell-based regenerative approach offers a durable and biologically compatible alternative to traditional HA-based skin boosters. This approach could represent a valuable complement to existing treatments for aging-related signs, potentially offering benefits for both aesthetic outcomes and psychological well-being. Further research involving larger, double-blinded studies with extended follow-up is required to validate these results and assess their long-term durability.

## Availability of data and materials

All data generated or analyzed during this study are included in this published article and its additional files.

## Funding

The author received no financial support for the research, authorship, and publication of this article.

## Ethical approval

The study protocol, which was the object of two university master's degrees titled "Plastic Aesthetic Surgery of Facial District" and “Regenerative Surgery and Medicine in Wound Care Management”, was approved with Rectoral Decree (D.R. n. 1794/2018) of 19 September 2018 and the Ethics on Research Committee of the School of Medicine, “Tor Vergata” University, Rome, Italy, with registration number #0031036/2018. The investigation protocol was developed in agreement with an associate professor contract #13489/2021.

## Patient consent

All patients received detailed oral and written information about the study, including the associated risks, benefits, and alternative therapies, and signed an informed consent form before any study procedures.

## Patient consent for photo publication

All patients received detailed oral and written information about the use of their pictures performed during pre- and post-operative follow-up. They signed an informed consent form before any study procedures, in which they authorized the picture's use for academic and scientific purposes.

## CRediT authorship contribution statement

**Pietro Gentile:** Validation, Methodology, Formal analysis, Writing – original draft.

## Declaration of competing interest

The author declared no potential conflicts of interest for the research, authorship, and publication of this article.

## Appendix Supplementary materials

Image, application 1

Image, application 2

Image, application 3
