# Supplementary material for: Autologous fibroblast therapy for facial rejuvenation: A randomized open-label controlled study
Source: JPRAS Open. 2026 Jun 1;50:632–44. doi: 10.1016/j.jpra.2026.05.045 (PMC13285675; doi:10.1016/j.jpra.2026.05.045)
Supplement: Supplementary file 1 [file mmc1.docx]

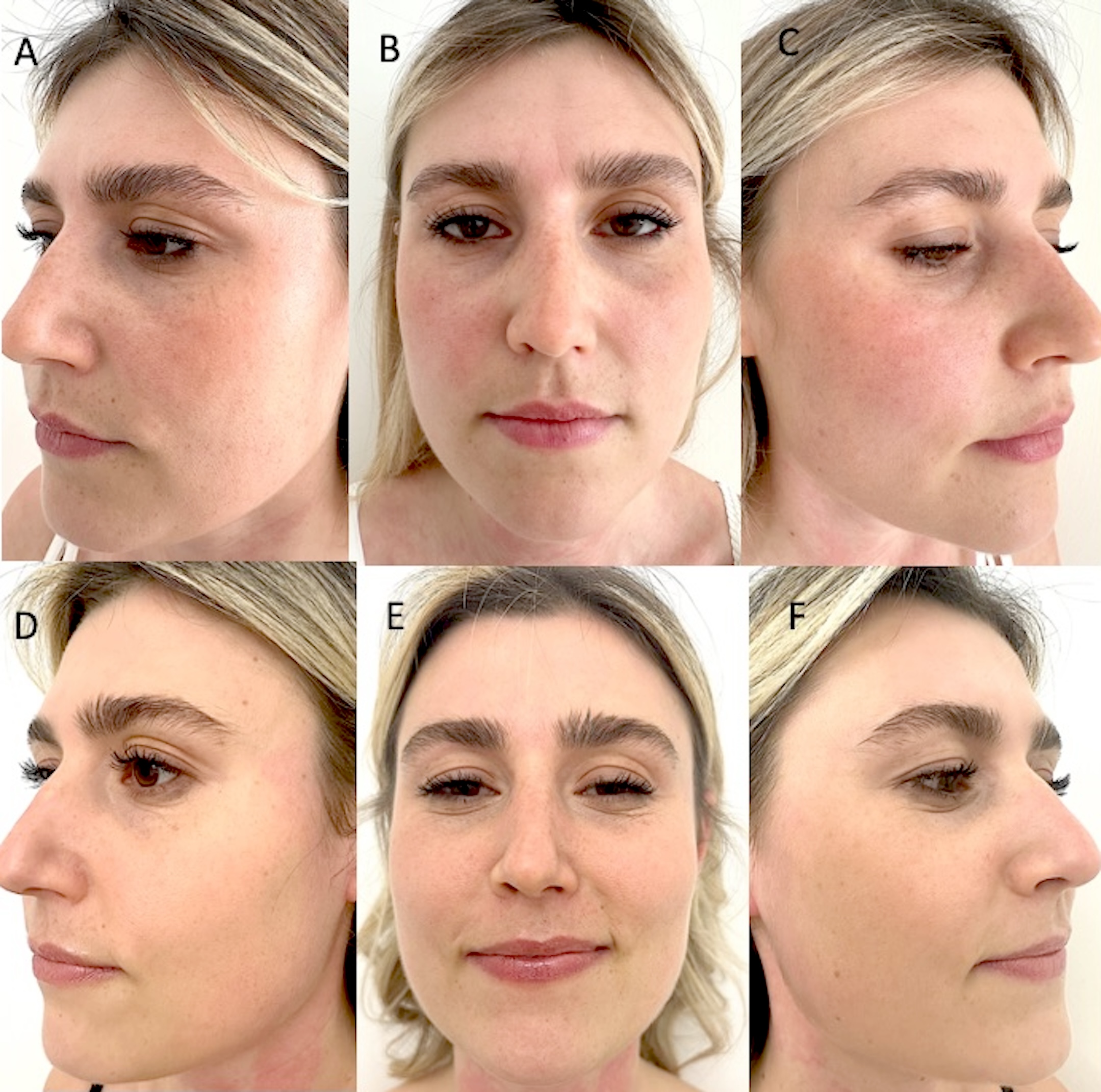
Supplemental Figure 1. A 28-year-old female affected by mild dermal thinning, loss of elasticity, and wrinkles, treated with a Hyaluronic Acid-based skin booster containing vitamins (NCTF-135HA, Filorga, Paris, France <https://fillmed.com/revitalize-nctf/>). A) Pre-operative projection in ¾ left view. B) Pre-operative projection in frontal view. C) Pre-operative projection in ¾ right view. D) Post-operative projection after 6 months (T3) in ¾ left view. Improvement of the skin texture, tissue elasticity, and brightness, with reduction of wrinkles. E) Post-operative projection in frontal view, evidencing all the improvements. F) Post-operative projection after 6 months in ¾ right view.


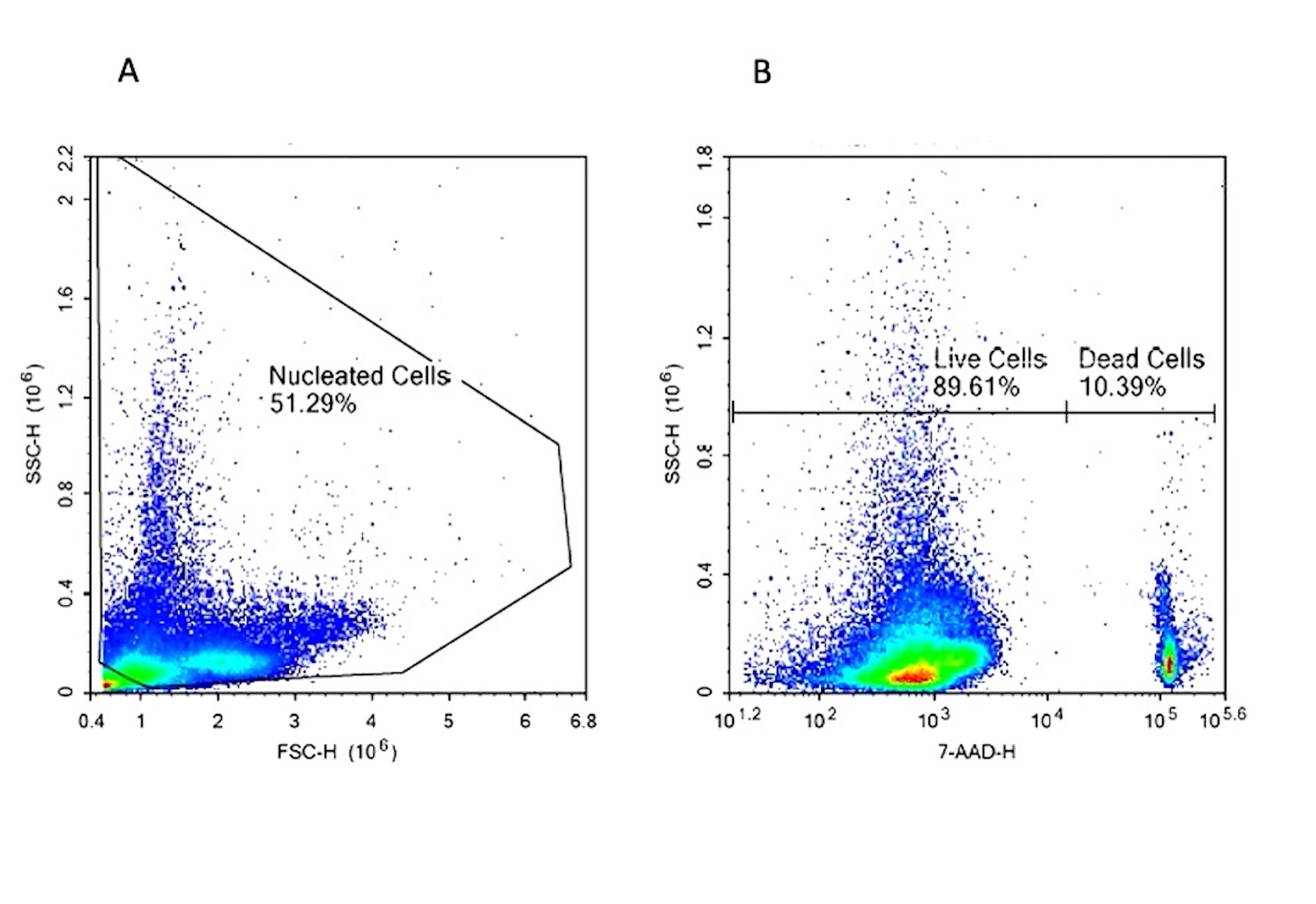
Supplemental Figure 2. Flow cytometer analysis. A) Flow cytometer analysis confirmed the presence of nucleated cells in A-FBs solution, documenting a concentration of 2.780.750 nucleated cells/ml (51.29%). B) The viability of the cells was 89.61% (live cells), while dead cells were 10.39%.

Supplemental Material 1. CONSORT 2025 checklist item description.

Supplemental Material 2 – Appendix A. Patient satisfaction questionnaire.
