# Supplementary material for: Autologous fibroblast therapy for facial rejuvenation: A randomized open-label controlled study
Source: JPRAS Open. 2026 Jun 1;50:632–44. doi: 10.1016/j.jpra.2026.05.045 (PMC13285675; doi:10.1016/j.jpra.2026.05.045)
Supplement: Supplementary file 2 [file mmc2.docx]

|  | Section/topic | No | CONSORT 2025 checklist item description | Reported on page no. |
| --- | --- | --- | --- | --- |
|  | **Title and abstract** | | |  |
|  | Title and structured abstract | 1a | The **Autologous Fibroblast Therapy for Facial Rejuvenation: A Randomized Open-Label Controlled Study** |  |
|  |  | 1b | **Background:** Facial aging is characterized by dermal thinning, loss of elasticity, and wrinkle formation. Autologous fibroblast transplantation has emerged as a promising regenerative technique.  **Objectives:** This study aims to assess the efficacy and safety of autologous fibroblasts for facial rejuvenation.  **Methods:** A randomized, open-label, controlled study was conducted. 40 participants aged 25–45 years with mild to moderate dermal thinning, loss of elasticity, and wrinkles were assigned to two groups: the study group (SG) treated with autologous fibroblasts (Am-FBs) and the control group (CG) treated with classic vitamins. Both groups received treatment at baseline (T0), at one month (T1), and three months (T2). Autologous fibroblasts were prepared according to the minimal manipulation rules using the Dermomine T-lab kit. Clinical outcomes were evaluated at T1, T2, six months (T3) and 12 months (T4) using the Wrinkle Severity Rating Scale (WSRS) and objective skin elasticity metrics.  **Results:** At 12 months, SG’s patients showed significant improvements in wrinkle depth and skin elasticity (*p* < 0.01) compared to the CG (*p* < 0.05). Patient satisfaction was higher in the SG. Adverse events were minimal and represented by transient erythema and edema in both SG and CG, with no major complications reported.  **Conclusions:** Am-FBs significantly improve signs of facial aging and present a safe option for skin rejuvenation. Further studies with larger sample sizes and longer follow-up are warranted. |  |
|  | **Open science** | | |  |
|  | Trial registration | 2 | The study protocol, which was the object of two university master's degrees titled "Plastic Aesthetic Surgery of Facial District" and “Regenerative Surgery and Medicine in Wound Care Management”, was approved with Rectoral Decree (D.R. n. 1794/2018) of 19 September 2018 and the Ethics on Research Committee of the School of Medicine, “Tor Vergata” University, Rome, Italy, with registration number #0031036/2018. The investigation protocol was developed in agreement with an associate professor contract #13489/2021. |  |
|  | Protocol and statistical analysis plan | 3 | All t-test analyses were performed using an online p-value calculator (<https://www.graphpad.com/quickcalcs/ttest1.cfm>) |  |
|  | Data sharing | 4 | Personal database of the author |  |
|  | Funding and conflicts of interest | 5a | The author received no financial support for the research, authorship, and publication of this article. |  |
|  |  | 5b | None |  |
|  | **Introduction** | | |  |
|  | Background and rationale | 6 | Facial aging is characterized by dermal thinning, loss of elasticity, and wrinkle formation. Autologous fibroblast transplantation has emerged as a promising regenerative technique. |  |
|  | Objectives | 7 | This study aims to assess the efficacy and safety of autologous fibroblasts for facial rejuvenation. |  |
|  | **Methods** | | |  |
|  | Patient and public involvement | 8 | Age 25-45 years, mild to moderate dermal thinning, loss of elasticity, wrinkles, signs of aging |  |
|  | Trial design | 9 | Randomized, open-label, case-controlled study. |  |
|  | Changes to trial protocol | 10 | None |  |
|  | Trial setting | 11 | Clinics, hospital. The trial was conducted in Rome, Italy. |  |
|  | Eligibility criteria | 12a | Age 20–50 years, patients with mild to moderate dermal thinning, loss of elasticity, wrinkles, facial soft tissue defects, facial skin aging, signs of facial aging |  |
|  |  | 12b | Plastic Surgeons |  |
|  | Intervention and comparator | 13 | Autologous Fibroblast cells (SG – Study group); Skin booster based on vitamines (CG- Control group) |  |
|  | Outcomes | 14 | *Clinical outcomes measures*  Primary Outcome:   - Improvement in wrinkle severity based on Wrinkle Severity Rating Scale (WSRS).   Secondary Outcomes:   - Dermal elasticity via Cutometer^®^ analysis and patient satisfaction surveys. - Patient-reported outcomes using a visual analog scale (VAS) for scar appearance and satisfaction. - Safety monitored via adverse event reporting and physical examination   Tertiary Outcomes:   - Information about the risks and side effects.   *Instrumental outcomes measures*   - *In vitro* presence of nucleated cells in the Am-FBs solution, - *In vitro* quantitative (cell amount) and qualitative (live and dead cells) analysis of the cell population contained in Am-FBs solution using a flow cytometer. - Fibroblast cell immunophenotyping. |  |
|  | Harms | 15 | Systematically |  |
|  | Sample size | 16a | 40 patients were enrolled after inclusion and exclusion criteria selection. An external collaborator generated a random allocation sequence and enrolled patients, as well as concealing  the sequence until the interventions were assigned. The author assigned the participants to interventions  according to randomization protocol. Demographic and medical information of the patients was recorded at the time of application. |  |
|  |  | 16b | As above mentioned. |  |
|  | Randomisation: |  |  |  |
|  | Sequence generation | 17a | An external collaborator generated a random allocation sequence and enrolled patients, as well as concealing  the sequence until the interventions were assigned. The author assigned the participants to interventions  according to randomization protocol. Demographic and medical information of the patients was recorded at the time of application. |  |
|  |  | 17b | The patient allocation sequence for both the SG and CG groups, based on the inclusion and exclusion criteria, was generated using an online randomization tool (https://www.randomizer.org) and was kept concealed by someone not involved in the trial management. The treatment allocation was known to the participants, study personnel, and outcome assessors. |  |
|  |  |  |  | **Reported on page no.** |
|  | Allocation concealment mechanism | 18 | Software, central computer |  |
|  | Implementation | 19 | The patient allocation sequence for both the SG and CG groups, based on the inclusion and exclusion criteria, was generated using an online randomization tool (https://www.randomizer.org) and was kept concealed by someone not involved in the trial management. The treatment allocation was known to the participants, study personnel, and outcome assessors. |  |
|  | Blinding | 20a | The patient allocation sequence for both the SG and CG groups, based on the inclusion and exclusion criteria, was generated using an online randomization tool (https://www.randomizer.org) and was kept concealed by someone not involved in the trial management. The treatment allocation was known to the participants, study personnel, and outcome assessors. |  |
|  |  | 20b | Not blinded |  |
|  | Statistical methods | 21a | T-tests and related p-value |  |
|  |  | 21b | All randomised participantsin SG and CG |  |
|  |  | 21c | None |  |
|  |  | 21d | None |  |
|  | **Results** | | |  |
|  | Participant flow, including flow diagram | 22a | N= 20 Study group and N=20 control Group |  |
|  |  | 22b | Both groups received treatment at baseline (T0), at one month (T1), and three months (T2). Autologous fibroblasts were prepared according to the minimal manipulation rules using the Dermomine T-lab kit. Clinical outcomes were evaluated at T1, T2, six months (T3) and 12 months (T4) using the Wrinkle Severity Rating Scale (WSRS) and objective skin elasticity metrics |  |
|  | Recruitment | 23a | N/A |  |
|  |  | 23b | N/A |  |
|  | Intervention and comparator delivery | 24a | The surgical procedures were performed by the author PG and all the patients enrolled were treated. |  |
|  |  | 24b | Am-FBs injections and Vitamins ingections |  |
|  | Baseline data | 25 | Table 1 |  |
|  | Numbers analysed,  outcomes and estimation | 26 | Between January 2024 and January 2025, 20 patients (study group - SG) affected by mild to moderate dermal thinning, loss of elasticity, and wrinkles were treated with Am-FBs. The SG was comprised of 10 females and 10 males aged 25–45 years Female pre-menopausal patients were 10 (100%). The author compared the results obtained with those of a control group (CG) made up of 20 patients treated with vitamins (NCTF) for the same above-mentioned reasons. The CG was comprised of 10 females and 10 males aged 25–45 years. Female pre-menopausal patients were 10 (100%). There were no significant differences in baseline characteristics between the two groups. All SG and CG patients underwent a full preoperative screening, including a detailed anamnesis (including patient expectations), complete clinical evaluation, and photographic and Wrinkle Severity Rating Scale (WSRS) assessment. Postoperative follow-up occurred at T1, T2, six months (T3) and 12 months (T4). Inclusion and exclusion selection criteria were described in Table 1. *Clinical assessment*  The procedures (Am-FBs) and (Vitamins) were successfully performed in all patients (SG and CG). The follow-up was performed after baseline (T0) at 1 month (T1), 3 months (T2), 6 months (T3) and 12 months (T4), All SG and CG patients were controlled at T4, concluding the follow-up.  *Primary Outcome*  At 12 months (T4), the SG treated with Am-FBs demonstrated a significant improvement in the WSRS score compared to the CG treated with Vitamins alone (mean improvement: 1.4 points in the SG vs. 0.3 points in the CG, *p* < 0.01).  *Secondary Outcomes*  Cutometer measurements showed a 27% increase in skin elasticity in SG patients, compared to a 5% increase in the CG patients (p < 0.01).  The SG exhibited greater reductions in wrikle visibility (mean change: 23% vs. 11%, *p* < 0.05) and higher patient satisfaction ratings (mean VAS score: 7.2 vs. 4.7, *p* < 0.05).  Adverse events were minimal, with no significant differences between the groups. The most common adverse event, reported by 3 SG patients and 3 CG patients respectively, was mild swelling and minor erythema at the injection sites, which resolved within 4 days.  *Tertiary Outcomes*  Satisfaction grade assessment questionnaire analysis showed that all people in both groups (SG and CG) would choose to undergo biorivitalization of the face, and they were sufficiently informed about the risks and complications of this procedure (including the risk to repeat the treatment more times and inefficacy of the procedures) |  |
|  | Harms | 27 | Adverse events were minimal, with no significant differences between the groups. The most common adverse event, reported by 3 SG patients and 3 CG patients respectively, was mild swelling and minor erythema at the injection sites, which resolved within 4 days. |  |
|  | Ancillary analyses | 28 | None |  |
|  | **Discussion** | | |  |
|  | Interpretation | 29 | This study supports previous research demonstrating the regenerative potential of Am-FBs for facial rejuvenation The intra-patient design controls for genetic and lifestyle factors, strengthening the findings. Our results align with long-term follow-up studies that report stable clinical improvement and no adverse tissue reactions. |  |
|  | Limitations | 30 | The four most significant limitations were a) the study's design as "open label", b) the custom-made approach, c) the small sample size, and d) the short follow-up. The ‘‘open-label’’ trial, instead of ‘‘single-blinded’’ or ‘‘double-blinded,’’ prevents having an objective evaluation, or, in any case, was not influenced by the knowledge of having undergone a treatment rather than another. This constitutes a study bias. On the other hand, in each case, the bias was limited by the ‘‘custom-made approach’’ for every patient, both for SG and CG. Respectively, the amount of the solution to inject was chosen based on the kind of defect. The relatively small sample size doesn’t permit a definitive description of the A-FB’s efficacy, not allowing the identification of any subgroups that could respond differently to the treatment. The limited follow-up based on a maximum of 12 months may not be enough to demonstrate the long-lasting results.  . |  |

Citation: Hopewell S, Chan AW, Collins GS, Hróbjartsson A, Moher D, Schulz KF, et al. CONSORT 2025 Statement: updated guideline for reporting randomised trials. BMJ. 2025; 388:e081123. <https://dx.doi.org/10.1136/bmj-2024-081123>
© 2025 Hopewell et al. This is an Open Access article distributed under the terms of the Creative Commons Attribution License (<https://creativecommons.org/licenses/by/4.0/>), which permits unrestricted use, distribution, and reproduction in any medium, provided the original work is properly cited.

*We strongly recommend reading this statement in conjunction with the CONSORT 2025 Explanation and Elaboration and/or the CONSORT 2025 Expanded Checklist for important clarifications on all the items. We also recommend reading relevant CONSORT extensions. See [www.consort-spirit.org](http://www.consort-spirit.org).
