# Supplementary material for: Autologous fibroblast therapy for facial rejuvenation: A randomized open-label controlled study
Source: JPRAS Open. 2026 Jun 1;50:632–44. doi: 10.1016/j.jpra.2026.05.045 (PMC13285675; doi:10.1016/j.jpra.2026.05.045)
Supplement: Supplementary file 3 [file mmc3.docx]

**Supplemental Material 2 - Appendix A. Patient’s satisfaction questionnaire.**

Degree of Satisfaction:

9 - Excellent;

8 - Very good;

7 – Good;

6 – Enough;

5 – Dissatisfied;

4 – Very Dissatisfied

In the pre-operative period

According to the scale above, grade questions 4 through 9 according to your degree of satisfaction:

1. How do you feel about your facial skin elasticity?

2. How do you feel about the visibility of your wrinkles?

3. How do you feel about your dermal thinning?

4. How do you feel about your loss of elasticity?

5. How do you feel about your facial skin lightness?

6. Did you have exhaustive information on risks and complications (including the risk of ineffective treatment and the possibility of repeating the treatment more times)?

In the post-operative period at 1 month (T1), 3 months (T2), 6 months (T3), and 12 months (T4).

According to the scale above, grade questions 4 through 9 according to your degree of satisfaction:

1. How do you feel about your facial skin elasticity?

2. How do you feel about your wrinkles reduction?

3. How do you feel about your dermal thinning improvement?

4. How do you feel about your loss of elasticity improvement?

5. How do you feel about your facial skin lightness improvement?

6. Did you have exhaustive information on risks and complications (including the risk of ineffective treatment and the possibility of repeating the treatment more times)?
